# The safety of preoperative carbohydrate drinks in extremely elderly patients assessed by gastric ultrasonography: a randomized controlled trial

**DOI:** 10.1186/s12871-024-02457-1

**Published:** 2024-02-26

**Authors:** Lingyang Chen, Nana Wang, Guohao Xie, Mingcang Wang, Yulong Yu, Huiqin Wang, Xiangming Fang

**Affiliations:** 1 Department of Anesthesiology, The First Affiliated Hospital, College of Medicine, Zhejiang University, Hangzhou, China; 2 Department of Anesthesiology, Taizhou Hospital of Zhejiang Province, Zhejiang University, Linhai, China

**Keywords:** Extremely elderly patients, Carbohydrate drink, Gastric ultrasonography, Gastric antral cross-sectional area

## Abstract

**Background:**

Modern perioperative guidelines encourage drinking oral carbohydrates 2 h before management. Nevertheless, research on the safety of preoperative carbohydrate drinks, particularly in extremely elderly patients is lacking. We aimed to evaluate the safety of carbohydrate drinks 2 h before surgery in extremely elderly patients (≥ 80 years) using gastric ultrasonography.

**Methods:**

We conducted a randomized prospective comparative study of 70 patients aged over 80 years who were scheduled for total knee arthroplasty, hip fracture or humerus fracture surgery. These patients were randomly assigned to the carbohydrate group (*n* = 35), which fasted from midnight, except for drinking 355 mL of a carbohydrate-containing fluid 2 h before surgery, or the fasting group (*n* = 35), which fasted from midnight and drank no fluid before surgery. The primary outcome of the study was the cross-sectional area (CSA) of the gastric antrum in the right lateral decubitus position (RLDP) before surgery. The secondary outcomes included CSA in the supine position, intraoperative blood glucose levels and their variability coefficients, Perlas grade, and the visual analog scale of subjective feelings.

**Results:**

The CSA in the RLDP and supine positions revealed no differences between the carbohydrate and fasting groups at 0 h preoperatively (*P* > 0.05). In the qualitative assessment, preoperative 0-h Perlas grading did not differ significantly between the groups (*P* > 0.05). From 2 h before surgery to transfer out of the post-anesthesia care unit, the average blood glucose level of patients in the carbohydrate group was significantly higher than that in the fasting group (*P* < 0.001) but remained within the normal range. Moreover, the blood glucose variability coefficient was significantly lower in the carbohydrate group than in the fasting group (*P* = 0.009). Oral intake of 355 mL carbohydrates before surgery significantly relieved patients’ feelings (*P* < 0.001).

**Conclusion:**

Preoperative consumption of carbohydrate drinks 2 h before surgery is safe in “healthy” extremely elderly patients. In addition, preoperative drinking has potential value in maintaining ideal blood glucose levels and stable blood glucose fluctuations perioperatively and improving subjective perceptions of preoperative preparation. This finding warrants further investigation in clinical practice.

**Trial registration:**

Chinese Clinical Trial Registry (Registration Number ChiCTR1900024812), first registered on 29/07/2019.

## Background

The purpose of fasting in selective surgeries is to reduce the risks of reflux and aspiration during the perioperative period [1, 2]. To ensure patients’ safety during the perioperative period, surgeons and anesthesiologists strongly recommend that patients scheduled for selective surgery begin fasting at midnight. However, owing to the uncertainty of surgery time, the actual fasting time for both selective and emergency surgeries is considerable for most patients, sometimes exceeding 12 h [3]. To prevent pulmonary aspiration occurs following regurgitation of gastric contents during induction period of anesthesia, blindly pursuing an extended fasting time for surgery patients fails to benefit patients and leads to adverse effects of varying degrees, such as decreased blood glucose levels [4], postoperative insulin resistance, increased metabolic stress after surgery, impaired tissue repair, and wound healing capacity [5]. In addition, long-term fasting significantly aggravates patient discomfort during preoperative preparation [6]. In recent years, an actual fasting time of > 10 h has been demonstrated to result in thirst, hunger, anxiety, and other adverse subjective feelings [7, 8]. The Enhanced Recovery After Surgery Society recommends preoperative oral carbohydrate therapy for selective surgery patients [9]. Clinical trials in several countries have demonstrated that oral intake of 800 mL of carbohydrates the night before surgery and 400 mL of carbohydrates 2 h before surgery is safe [10, 11]. This therapy can reduce postoperative insulin resistance, decrease protein consumption, and minimize postoperative complications [10].

With societal development and the overall increase in the average human lifespan, elderly patients undergoing surgical treatment have become a common phenomenon in clinical practice [12, 13]. Some evidence suggests that as age increases, the gastric emptying speed in elderly individuals may decrease [14–16]. Moreover, the increase of gastric volume(GV) caused by delayed gastric emptying is a high risk factor for aspiration. Ultrasound measurement of the cross-sectional area(CSA) is a commonly considered as an useful and effective method for evaluating gastric emptying [17–20]. The CSA of the antrum can reflect the whole volume of gastric content and showed a remarkably positive correlation between the CSA and GV [21]. Therefore, we aimed to conduct a prospective randomized controlled trial to assess the safety of carbohydrate drinks intake 2 h before surgery in extremely elderly patients (≥ 80 years) through gastric ultrasonography.

## Methods

### Study methods and patient cohort

This trial was approved by the Ethics Review Committee of Taizhou Hospital of Zhejiang Province (K20190752) and registered in the Chinese Clinical Trial Registry (https://www.chictr.org.cn/showproj.html?proj=41377), first registered on 29/07/2019, under the registration number ChiCTR1900024812. We used the CONSORT checklist when writing our report [22]. Informed consent was obtained from all patients or their close relatives before their participation. This study complied with the 1964 Declaration of Helsinki and its subsequent amendments.

This prospective randomized controlled trial was conducted in patients scheduled for selective total knee arthroplasty, hip fracture or humerus fracture surgery at Taizhou Hospital, Zhejiang Province, from August 2020 to August 2022. The inclusion criteria were: (1) Patients aged ≥ 80 years planning to undergo selective total knee arthroplasty, hip fracture, or humerus fracture surgery; (2) American Society of Anesthesiologists (ASA) grade 1–3; (3) NYHA class I-II; (4) Body mass index: 18–27 kg/m^2^; (5) Signed informed consent form. The exclusion criteria were: (1) Patients undergoing surgery for fractures other than the aforementioned three types; (2) Patients with a history of gastrointestinal surgery, gastroesophageal reflux, gastrointestinal obstruction, or related medical history; (3) Patients with diabetes or impaired glucose tolerance; (4) Patients with severe organ dysfunction; (5) Patients with cognitive impairment or neurological diseases that hindered their participation in the study; and (6) Patients who refused to participate in the study. A total of 70 patients aged ≥ 80 years were finally included according to the inclusion and exclusion criteria set by the study.

### Randomization and blinding

Patients were randomized using a computer-generated randomization sequence performed by an investigator who was not involved in patient care. The 70 elderly patients were allocated into two groups: one group was assigned as the fasting group (*n* = 35), and the other group was assigned as the carbohydrate group (*n*=35). In the fasting group, the patients started fasting at midnight before surgery. The carbohydrate group also started fasting from midnight before surgery, except for receiving 355 mL carbohydrate drinks (Outfast® [14.2% carbohydrate, 238 kcal*100 mL^− 1^,280–300 mmol/L]; HUMANWELL FSMP, Hubei Province, China) 2 h before surgery. The investigators of the study (including the ultrasound-evaluating physicians and visual analog scale (VAS) assessors) were unaware of the specific group assignments. All surgeries were performed by the same orthopedic team who were not involved in the study and were blinded to the group allocation. Random number allocations were performed by the investigator (HQ Wang.), who informed the patients how to fast before surgery according to enrollment. Assessments of study outcomes were performed by investigators (LY Chen, NN Wang and YL Yu) blinded to group allocation.

### Gastric ultrasonography

All patients underwent ultrasound examination in the preoperative preparation room was performed 2 h before the surgery (prior to drinking carbohydrates) and 5 min before the start of the surgery to assess the patient’s gastric antrum CSA in the supine and right lateral decubitus positions (RLDP). Each ultrasound examination was conducted independently by the same anesthesia specialist, and the average of three measurements was taken as the measurement result. A curvilinear array low-frequency transducer (2–5 MHz) and LogiQ E ultrasound equipment (GE Healthcare, Piscataway, NJ, USA) were used in a standard abdominal setting [23].

**Fig. 1:**
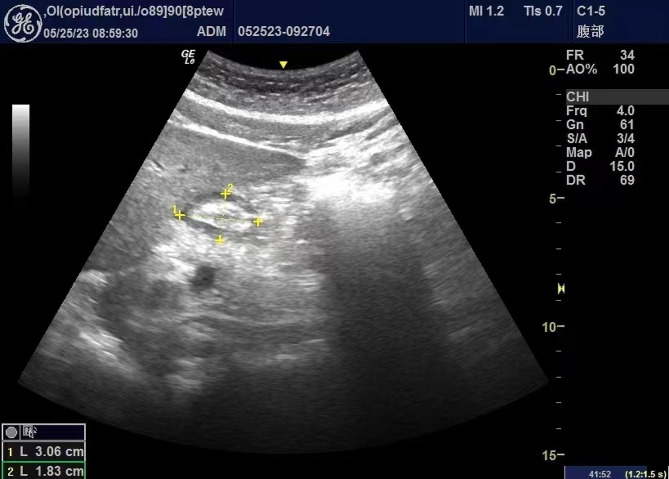
Representative figure of gastric CSA measurement, showing CSA measurements in the 2 perpendicular diameters. (Line1: LD; Line2: SD). Abbreviations: CSA, cross-sectional area; LD, longest diameter, SD, shortest diameter

Gastric ultrasound examinations include qualitative and quantitative evaluations. In qualitative evaluation, the gastric antrum is defined as “empty” when it is flat without fluid. When fluid is detected, the gastric antrum is defined as “fluid-filled.” Based on qualitative evaluation, a semi-quantitative Perlas grading method is used to classify the gastric antrum into three levels [19]: Grade 0 (low risk): gastric antrum ultrasound scan in the supine position and RLDP shows no fluid; Grade 1 (moderate risk): gastric antrum ultrasound scan in the supine position shows no fluid, but fluid is present in the RLDP; Grade 2 (high risk): gastric antrum ultrasound scan in both positions shows fluid. For quantitative evaluation, two vertical diameters are measured: the longest diameter (LD, cm) and shortest diameter (SD,cm) (Fig. 1). The CSA of the gastric antrum is calculated using the formula CSA (cm^2^) = (LD × SD × π)/4 [24].

### Assessment outcomes

The primary outcome was the CSA of gastric antrum in the RLDP before surgery. The secondary outcome was the CSA in the supine position. Other secondary outcomes were also collected and recorded, including intraoperative aspiration, average blood glucose (GLUave), blood glucose variation coefficient (GLUcv) from 2 h before surgery to the end of surgery (GLUcv reflects the fluctuation of blood glucose within a certain period, calculated as GLUcv = GLUsd × 100/GLUave) [25], and the occurrence of perioperative hypoglycemia (hypoglycemia was defined as glycemia < 3.0 mmol/L) [26]. VAS was used to evaluate the degree of satiety, thirst, and weakness. The scale scores range from 0 (none) to 10 (extreme) [27]. Satiety was divided into three levels: 0–3 represents no satiety, 4–6 represents moderate satiety, and 7–10 represents significant bloating. Thirst was divided into three levels: 0–3 represents no thirst, 4–6 represents tolerable thirst, and 7–10 represents significant thirst that cannot be tolerated. Weakness is divided into three levels: 0–3 represents no weakness, 4–6 represents the ability to climb two flights of stairs, and 7–10 represents weakness that made climbing stairs impossible.

### Sample size calculation

This was a parallel, randomized, controlled study. The intervention group comprised the carbohydrate group, and the control group comprised the fasting group. The study was designed based on preliminary findings, which have not been previously published. Specifically, our team conducted a preliminary experiment involving 137 participants categorized into young and elderly groups. The objective was to assess dynamic gastric ultrasound within two hours after carbohydrate ingestion. The unpublished results revealed a 30% difference was observed in gastric content volume in elderly patients after oral administration of 355 mL carbohydrate-containing fluid 2 h before surgery, with 1.05 mL/kg in the elderly group. With α = 0.05 (two-sided) and β = 0.20, assuming a dropout rate of 10% among study participants, an estimated sample size of 35 cases per group was anticipated. In the actual study, 35 cases were included in each group.

### Statistical analysis

This study primarily utilized SPSS statistical software version 26.0 (IBM Corp., Armonk, NY) for data processing and analysis. The Kolmogorov–Smirnov test was used to verify the normal distribution of continuous variables. Variables conforming to the normal distribution were represented using the mean ± standard deviation and analyzed using the Student’s t-test. For those not conforming to the normal distribution, the median (interquartile range) was used as the representation, and the analysis was conducted using the Mann–Whitney U test. Categorical variables were expressed as numbers (percentages), and comparative analyses were performed using the chi-square test or Fisher’s exact test. All *P* values were considered significant at a level of 0.05.

## Results

**Fig. 2:**
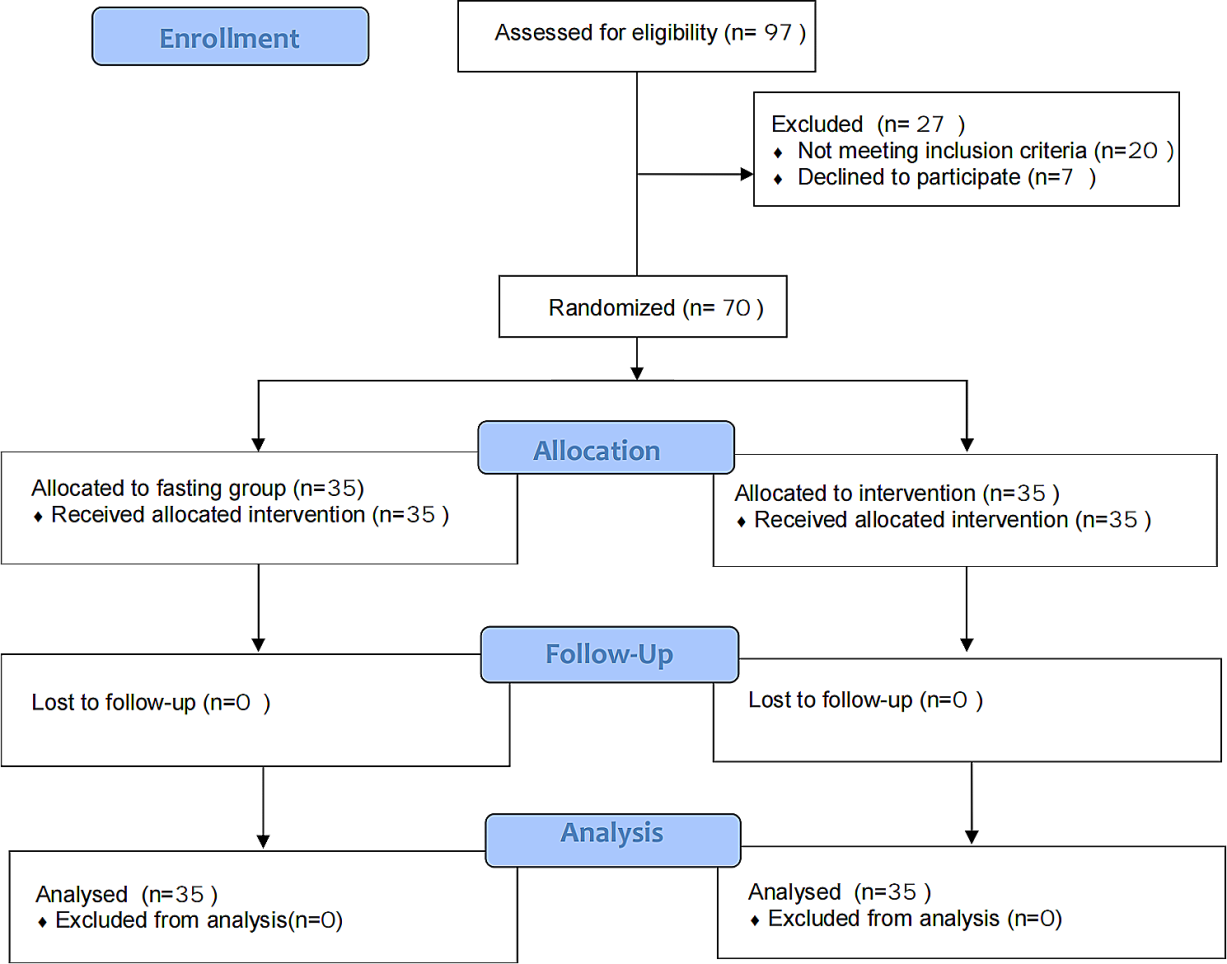
Patient inclusion flow chart. Abbreviations: VAS, visual analogue scale

We conducted a qualification review of 97 patients aged over 80 years who planned to undergo selective total knee arthroplasty, hip fracture, or humerus fracture surgery at Taizhou Hospital of Zhejiang Province from August 2020 to August 2022. Based on the inclusion and exclusion criteria of the study, 10 patients with concomitant diabetes or impaired glucose tolerance, six patients who had previously undergone gastric surgery or had gastroesophageal reflux disease, four patients with cognitive impairments, and seven patients who disagreed to participate in the study were excluded. Ultimately, 70 patients were included in this study (Fig. 2). The fasting and carbohydrate groups comprised 35 patients.

### Comparison of baseline characteristics between the two groups

As presented in Table 1, apart from the time of liquid fasting, no differences were observed between the two groups in demographic characteristics (age, sex, height, weight, and body mass index), ASA classification, duration of solid food fasting, type of surgery, and gastric ultrasound examination results 2 h before surgery (CSA, Perlas classification) (*P* > 0.05). The median liquid fasting time for patients in the fasting group was 11.31 h, whereas that for patients in the carbohydrate group was 2 h (*P* < 0.001).

**Table 1:** Comparison of baseline parameters between two groups

	Fasting group (*n* = 35)	Carbohydrate group (*n* = 35)	*P* Value
Age (year)	84(82–87)	84(81–86)	0.269
Sex(male/female)	15(42.85%)/20(57.15%)	17(48.57%)/18(51.43%)	0.631
Weight (kg)	53.0(50.0–58.0)	55.0(48.0–55.0)	0.995
Height (cm)	156.0(151.0-167.0)	162.0(153.0-167.0)	0.167
BMI (kg/m^2^)	21.93(21.03–22.64)	21.79(19.65–23.10)	0.267
ASA physical status(II/III)	22(62.85%)/13(37.15%)	20(57.15%)/15(42.85%)	0.626
Fasting hours for solids(h)	14.28(13.70-14.81)	14.06(13.19–14.88)	0.404
Fasting hours for liquids(h)	11.31(10.87–12.08)	2.00(2.00–2.00)	<0.001
Type of surgery			
Hip fracture surgery	16(45.71%)	14(40.00%)	0.494
Total knee arthroplasty	10(28.58%)	9(25.71%)	
Humerus fracture surgery	9(25.71%)	12(34.29%)	
CSA, RLDP, 2 h before surgery (cm^2^)	5.86(5.13–6.69)	5.52(4.70–6.91)	0.466
CSA, supine, 2 h before surgery (cm^2^)	3.73(3.41–4.49)	3.83(3.24–4.72)	0.944
Perlas grade 0, 2 h before surgery	26(74.28%)	27(77.14%)	0.727
Perlas grade 1, 2 h before surgery	8(22.86%)	6(17.14%)	
Perlas grade 2, 2 h before surgery	1(2.86%)	2(5.72%)	

Abbreviations: BMI, body mass index; CSA, cross-sectional area; RLDP, right lateral decubitus position

### Comparison of objective outcomes between the two groups

Table 2 presents the comparison of objective outcomes between the two groups. The median preoperative 0-h CSA in RLDP was 6.14 (5.24–6.54) cm^2^ in the carbohydrate group and 5.86 (5.13–6.69) cm^2^ in the fasting group, revealing no difference (*P* > 0.05). No significant difference was observed in the preoperative 0-h CSA when measured in the supine position between the two groups (*P* > 0.05) (Table 2).

**Table 2:** Comparison of endpoint indicators between two groups

	Fasting group (*n* = 35)	Carbohydrate group (*n* = 35)	*P* Value
**Primary measurement**			
CSA, RLDP, 0 h before surgery (cm^2^)	5.86(5.13–6.69)	6.14(5.24–6.54)	0.353
**Secondary measurements**			
CSA, supine, 0 h before surgery (cm^2^)	4.46(3.79–5.22)	4.00(3.56–5.04)	0.557
Perlas grade 0, 0 h before surgery	23(65.71%)	24(68.57%)	0.964
Perlas grade 1, 0 h before surgery	10(28.57%)	9(25.71%)	
Perlas grade 2, 0 h before surgery	2(5.71%)	2(5.71%)	
Aspiration in surgery	0(0.0%)	0(0.0%)	1.000
GLUave (mmol/L)	6.42(6.11–6.56)	7.99(7.30–8.49)	<0.001
GLUcv	22.56(19.61–27.57)	16.71(10.71–24.32)	0.009
Hypoglycemia during surgery	17(6.1%)2（5.71%）	0（0%）	0.493

Abbreviations: CSA, cross-sectional area; RLDP, right lateral decubitus position, GLUave, average blood glucose; GLUcv, blood glucose variation coefficient

In the qualitative assessment, no statistical difference was observed in the preoperative 0-h Perlas grading between the two groups. Both groups had two patients (5.7%) with Perlas grade 2, indicating a high risk. No incidents of intraoperative aspiration occurred in either group.

### Blood glucose comparison between the two groups

During the period from 2 h before surgery to transfer out of the post-anesthesia care unit, the carbohydrate group had significantly higher average blood glucose levels (7.99 (7.30–8.49) mmol/L) compared with the fasting group (6.42 (6.11–6.56) mmol/L) (*P* < 0.001) but remained within normal range. The patients in the carbohydrate group had significantly lower blood glucose variability compared with the fasting group (16.71 (10.71–24.32) vs. 22.56 (19.61–27.57), *P* = 0.009). Notably, no hypoglycemic events (< 3.0 mmol/L) occurred intraoperatively in the carbohydrate group, whereas the fasting group experienced two such instances; however, the incidence rates between the two groups did not differ significantly (*P* = 0.493).

### Comparison of subjective feelings between the two groups

**Fig. 3:**
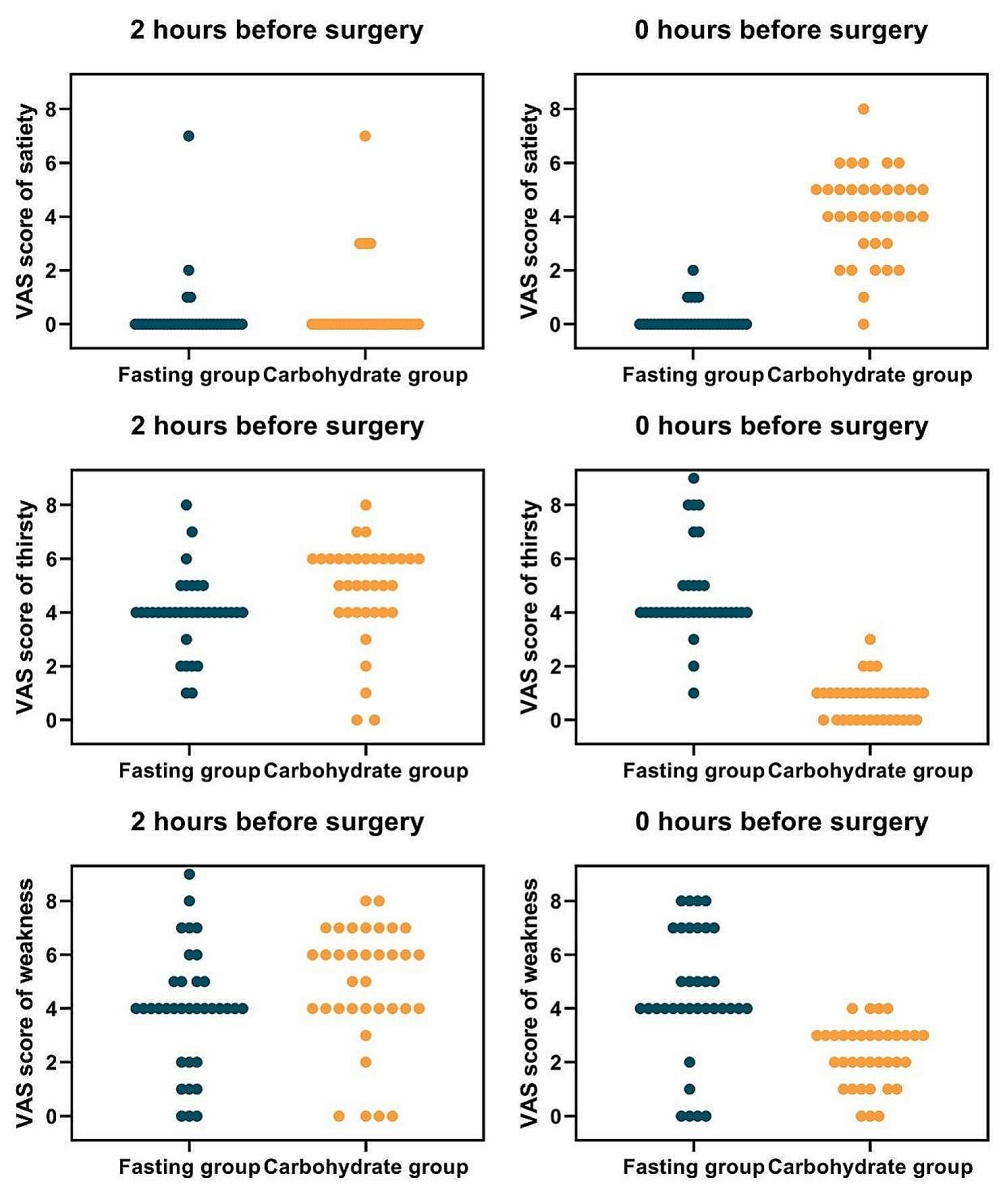
Comparison of visual analogue scale (VAS) score of different subjective sensations between two groups

Table 3; Fig. 3 present the VAS scores of the preoperative 2-h subjective feelings (satiety, thirst, and weakness) in the two groups. Two hours before surgery, no difference was observed in the VAS scores for satiety, thirst, and weakness between the two groups. Approximately 97% of patients felt no satiety, approximately 80% experienced moderate to severe thirst, and more than 70% experienced moderate to severe weakness in the fasting group.

**Table 3:** Comparison of subjective sensations between two groups

	2 h before surgery		*P* Value	0 h before surgery		*P* Value
	Fasting group(*n* = 35)	Carbohydrate group(*n* = 35)		Fasting group(*n* = 35)	Carbohydrate group(*n* = 35)	
**Satiety**: “Do you feel full?”						
None (0–3)	34(97.1%)	34(97.1%)	1.000	35(100.0%)	10(28.6%)	<0.001
Moderate (4–6)	0(0.0%)	0(0.0%)		0(0.0%)	24(68.6%)	
Obvious (7–10)	1(2.9%)	1(2.9%)		0(0.0%)	1(2.9%)	
**Thirsty**: “Do you want to drink water?”						
None (0–3)	7(20.0%)	5(14.3%)	0.840	3(8.6%)	35(100.0%)	<0.001
Moderate: can tolerate (4–6)	26(74.3%)	27(77.1%)		26(74.3%)	0(0.0%)	
Obvious: can’t tolerate (7–10)	2(5.7%)	3(8.6%)		6(17.1%)	0(0.0%)	
**Weakness**: “Do you feel weak?”						
None (0–3)	9(25.7%)	6(17.1%)	0.460	6(17.1%)	31(88.6%)	<0.001
Moderate: can climb two floors (4–6)	21(60.0%)	20(57.1%)		19(54.3%)	4(11.4%)	
Obvious: can’t climb two floors (7–10)	5(14.3%)	9(25.7%)		10(28.6%)	0(0.0%)	

At the start of surgery, significant differences were observed in the VAS scores for satiety, thirst, and weakness between the two groups. All patients (100%) in the fasting group felt no satiety and wanted to eat, whereas 68.6% of patients in the carbohydrate group reported moderate satiety after receiving 355 mL of carbohydrates. Compared with 2 h before surgery, the proportion of patients with moderate-to-severe thirst in the fasting group increased from 80% to 91.4% at the beginning of surgery, whereas all patients in the carbohydrate group experienced no obvious thirst. Compared with 2 h before surgery, the proportion of patients with moderate-to-severe weakness in the fasting group increased from 74.3% to 82.9% at the beginning of the operation, whereas the proportion of patients with moderate-to-severe weakness in the carbohydrate group decreased from 82.8% to 11.4%.

## Discussion

Prolonged preoperative fasting and fluid restriction have been proven to be more harmful than beneficial, especially in frail elderly populations [6, 14, 16, 28]. However, research on the safety of consuming carbohydrate fluids 2 h before surgery in elderly individuals, particularly those over 80 years of age, is lacking, resulting in insufficient evidence to determine whether fasting and drinking periods should be shortened for this age group. The hypothesis that the high risk of preoperative oral carbohydrate intake in elderly patients has made it difficult to conduct such studies in the elderly population. In recent years, gastric ultrasound examination has been considered an effective and safe method for evaluating gastric emptying by measuring the CSA of gastric antrum [17–19]. In our study, we assessed the safety of carbohydrate drinks 2 h before surgery compared with midnight fasting in extremely elderly patients (≥ 80 years) using gastric ultrasound. We observed no significant difference in the CSA measured in the RLDP at 0 h before surgery between the two groups. The Perlas grading before surgery did not differ between the two groups in our study. The satiety, thirst, and weakness scores improved, and more stable blood glucose levels were observed after ingestion of the carbohydrate drink.

The aspiration of gastric contents into the lungs is one of the most dangerous complications of anesthesia [1, 29, 30]. It was reported to be the leading cause of anesthesia-related deaths in France in 1999 [31]. The severity of aspiration and its clinical consequences mainly depend on the volume of aspirated gastric contents [32]. Therefore, a simple, noninvasive, and rapid method is necessary to determine the volume of gastric contents to help doctors assess the risk of aspiration, especially in elderly patients. Gastric point of care ultrasound can provide high reliability to evaluat nature and volume of gastric content perioperative period. The CSA of antrum can reflect the volume of gastric content [17]. Currently, three body positions are used to measure CSA. However, the RLDP is considered the best position for measurment of gastric content, because the antrum is at the lowest point of the stomach in this position. The CSA measured in RLDP correlated strongly with GV [33]. We measured and analyzed the CSA of the gastric antrum in the RLDP before surgery as the primary outcome measure, which is the most important factor for calculating gastric volume, based on the formula described by Perlas et al. [34]. Furthermore, GV is an important factor for estimating the severity of aspiration and regurgitation. In this study, we used gastric ultrasonography to evaluate CSA and compared the incidence of aspiration during surgery to assess the safety of preoperative carbohydrate intake in elderly patients. The results of this study revealed no significant differences in the preoperative 0-h CSA measured in both the RLDP and supine positions between the two groups. In the qualitative evaluation, no significant difference was observed in Perlas grading before surgery between the two groups. Moreover, none of the 70 patients experienced aspiration during surgery. These results demonstrated the safety of preoperative carbohydrate intake in elderly patients (≥ 80 years old). This aligns with previous reports demonstrating that ingestion of carbohydrates 2 h before surgery did not delay gastric emptying or increase the residual gastric volume [35, 36].

Long-term fasting and drinking abstinence can cause a decrease in blood glucose levels, a decrease in insulin secretion, and an increase in glucagon secretion. This promoted the decomposition of proteins, fats, and glycogen [4]. Furthermore, prolonged fasting before surgery can induce insulin resistance, affect tissue repair and wound healing, and reduce the body’s ability to resist infections [5]. Therefore, performing invasive surgery under the stress of prolonged fasting and abstinence from drinking can lead to hemodynamic disorders, collapse, and shock. Simultaneously, advanced age is a pivotal etiological factor in the development of hypoglycemia, as highlighted by Tourkmani et al. [37], who identified the geriatric cohort as a distinct population prone to hypoglycemic incidents. Investigations have revealed that for every decade of life, the risk of hypoglycemia increases by 11%. This elevated risk appears to be associated with polypharmacy in elderly individuals, diminished drug clearance, and sluggish feedback regulatory responses. Consequently, meticulous attention is warranted toward monitoring the perioperative glycemic status in the geriatric populace [38]. Previous clinical trials have demonstrated that the oral consumption of 800 mL of carbohydrates the night before surgery and 400 mL of carbohydrates 2 h before surgery could reduce postoperative insulin resistance, decrease protein consumption, and reduce postoperative complications. This study also reached similar conclusions [10]: oral consumption of carbohydrates 2 h before surgery could lead to ideal blood glucose levels and stable blood glucose fluctuations during the perioperative period. A retrospective cohort study conducted by Lee revealed that long-term glucose variability demonstrated a positive correlation with the risk of stroke, myocardial infarction, and all-cause mortality in diabetes [39]. Another study revealed that with an enhancement of the blood glucose fluctuations and the inflammatory response increased, blood glucose variability was correlated with early neurological improvement in patients with acute ischemic stroke [40]. Therefore, we speculate that lower blood glucose variability is beneficial for early recovery in elderly patients after the intake of carbohydrates (355 mL 2 h before surgery).

In recent years, clinical findings have revealed that preoperative fasting for more than 10 h and abstaining from drinking for more than 6 h can lead to adverse reactions such as thirst, hunger, anxiety, and dehydration in patients [8]. A randomized, double-blind trial revealed that the probability of nausea and vomiting was significantly reduced in patients who took 400 mL carbohydrates orally 2 h before surgery compared with patients who fasted for 8 h [10]. Continuous attempts have been made to reduce patient discomfort during the perioperative period and increase patient satisfaction. To ensure that the patients were in good physical condition before surgery, researchers administered oral glucose- or carbohydrate-containing drinks to the patients prior to surgery. This approach significantly alleviated patient discomfort, such as thirst and hunger [41–43]. In this study, we discovered that prolonged fasting and abstinence induced patient discomfort, as assessed by the VAS scores of satiety, thirst, and weakness 2 h before surgery. We observed lower VAS scores for thirst and fatigue in the carbohydrate group than in the fasting group at 0 h before surgery, which aligns with previous reports ingestion of carbohydrates before surgery effectively reduced patients’ thirst and weakness [41–43]. However, in this study, VAS scores for satiety were significantly higher in the carbohydrate intake group than in the fasting group. . Furthermore, these findings suggest that preoperative carbohydrate intaking is feasible.

This study has several limitations. First, this study was limited to patients undergoing orthopedic surgery and requires validation in other populations, such as the elderly population undergoing gastrointestinal surgery. Second, this was a single-center study, and the homogeneity of the sample may reduce the generalizability of the results. In future, multicenter collaborative research is needed to validate these findings. Finally, this study excluded patients with diabetes and impaired glucose tolerance, and further research is needed to explore fasting and dietary strategies for these patients.

## Conclusions

Using gastric ultrasonography, this study demonstrates that preoperative oral carbohydrate intake is safe in “healthy” patients aged 80 years and above and does not increase the incidence of intraoperative aspiration. Moreover, it provides ideal control of blood glucose levels and stabilizes blood glucose fluctuations during the perioperative period. In addition, this protocol can significantly improve the subjective comfort of elderly patients during preoperative preparation and should be implemented in clinical practice.

## Data Availability

The datasets used during the current study are available from the corresponding author on reasonable request.

## Abbreviations

ASA: American Society of Anesthesiologists
VAS: Visual analog scale
CSA: Cross-sectional area
RLDP: Right lateral decubitus position
LD: Longest diameter
SD: Shortest diameter
GLUave: Average blood glucose
GLUcv: Blood glucose variation coefficient
GV: Entire volume

## References

[1] Brady M, Kinn S, Stuart P. Preoperative fasting for adults to prevent perioperative complications. Cochrane Database Syst Rev 2003(4):Cd004423.10.1002/14651858.CD00442314584013

[2] Salem MR, Gaucher D, Joseph NJ (2005). Adequate preoperative fasting and aspiration: factors affecting regurgitation. Anesthesiology.

[3] Falconer R, Skouras C, Carter T, Greenway L, Paisley AM (2014). Preoperative fasting: current practice and areas for improvement. Updates Surg.

[4] Awad S, Lobo DN (2012). Metabolic conditioning to attenuate the adverse effects of perioperative fasting and improve patient outcomes. Curr Opin Clin Nutr Metab Care.

[5] Bragazzi NL, Sellami M, Salem I, Conic R, Kimak M, Pigatto PDM, Damiani G. Fasting and its impact on skin anatomy, physiology, and Physiopathology: a Comprehensive Review of the literature. Nutrients 2019;11(2). 10.3390/nu11020249 PMC6413166 30678053

[6] Søreide E, Ljungqvist O (2006). Modern preoperative fasting guidelines: a summary of the present recommendations and remaining questions. Best Pract Res Clin Anaesthesiol.

[7] Fawcett WJ, Thomas M (2019). Pre-operative fasting in adults and children: clinical practice and guidelines. Anaesthesia.

[8] Noba L, Wakefield A (2019). Are carbohydrate drinks more effective than preoperative fasting: a systematic review of randomised controlled trials. J Clin Nurs.

[9] Ljungqvist O, Scott M, Fearon KC (2017). Enhanced recovery after surgery: a review. JAMA Surg.

[10] Nygren J, Thorell A, Ljungqvist O (2015). Preoperative oral carbohydrate therapy. Curr Opin Anaesthesiol.

[11] Tewari N, Awad S, Duska F, Williams JP, Bennett A, Macdonald IA, Lobo DN (2019). Postoperative inflammation and insulin resistance in relation to body composition, adiposity and carbohydrate treatment: a randomised controlled study. Clin Nutr.

[12] Goncalves TJM, Goncalves S, Nava N, Jorge VC, Okawa AM, Rocha VA, Forato LCH, Furuya VAO, Martins SS, Oksman D (2021). Perioperative Immunonutrition in Elderly patients undergoing total hip and knee arthroplasty: impact on postoperative outcomes. JPEN J Parenter Enter Nutr.

[13] Edelstein AI, Dillingham TR, McGinley EL, Pezzin LE (2023). Hemiarthroplasty Versus Total hip arthroplasty for femoral Neck fracture in Elderly patients: twelve-Month risk of revision and dislocation in an Instrumental Variable Analysis of Medicare Data. J Bone Joint Surg Am.

[14] Brogna A, Ferrara R, Bucceri AM, Lanteri E, Catalano F (1999). Influence of aging on gastrointestinal transit time. An ultrasonographic and radiologic study. Invest Radiol.

[15] Soenen S, Rayner CK, Horowitz M, Jones KL (2015). Gastric emptying in the Elderly. Clin Geriatr Med.

[16] Madsen JL, Graff J (2004). Effects of ageing on gastrointestinal motor function. Age Ageing.

[17] Van de Putte P, Perlas A (2014). Ultrasound assessment of gastric content and volume. Br J Anaesth.

[18] Chang JE, Kim H, Won D, Lee JM, Jung JY, Min SW, Hwang JY (2020). Ultrasound assessment of gastric content in fasted patients before elective laparoscopic cholecystectomy: a prospective observational single-cohort study. Can J Anaesth = J Canadien D’anesthesie.

[19] Perlas A, Davis L, Khan M, Mitsakakis N, Chan VW (2011). Gastric sonography in the fasted surgical patient: a prospective descriptive study. Anesth Analg.

[20] Dhanger S, Vaidhyanathan B, Joseph Raajesh IJ (2022). Pre-anaesthetic sonographic assessment of gastric antrum in parturient scheduled for elective caesarean section - A prospective cross-sectional study. Indian J Anaesth.

[21] Bouvet L, Mazoit JX, Chassard D, Allaouchiche B, Boselli E, Benhamou D (2011). Clinical assessment of the ultrasonographic measurement of antral area for estimating preoperative gastric content and volume. Anesthesiology.

[22] Schulz KF, Altman DG, Moher D, Group C (2010). CONSORT 2010 statement: updated guidelines for reporting parallel group randomised trials. BMJ.

[23] Cubillos J, Tse C, Chan VW, Perlas A (2012). Bedside ultrasound assessment of gastric content: an observational study. Can J Anaesth = J Canadien D’anesthesie.

[24] Cho EA, Kim MS, Cha YB, Lee MS, Song T. Evaluation of Gastric Emptying Time of a Rice-Based Meal Using Serial Sonography. BioMed research international. 2019;2019:5917085. 10.1155/2019/5917085 PMC6855072 31781625

[25] Sattar N, Preiss D (2017). Research digest: hypoglycaemia and glucose variability. Lancet Diabetes Endocrinol.

[26] Cryer PE, Axelrod L, Grossman AB, Heller SR, Montori VM, Seaquist ER, Service FJ, Endocrine S (2009). Evaluation and management of adult hypoglycemic disorders: an endocrine Society Clinical Practice Guideline. J Clin Endocrinol Metab.

[27] Faiz KW. [VAS–visual analog scale]. Tidsskrift den Norske Laegeforening: Tidsskrift Praktisk Medicin ny Raekke. 2014;134(3):323. 10.4045/tidsskr.13.1145 24518484

[28] Nygren J (2006). The metabolic effects of fasting and surgery. Best Pract Res Clin Anaesthesiol.

[29] Gardner AM, Pryer DL (1966). Historical and experimental study of aspiration of gastric and oesophageal contents into the lungs in anaesthesia. Br J Anaesth.

[30] Sanivarapu RR, Gibson J. Aspiration Pneumonia. In: *StatPearls* edn. Treasure Island (FL) with ineligible companies. Disclosure: Joshua Gibson declares no relevant financial relationships with ineligible companies.: StatPearls Publishing Copyright © 2023, StatPearls Publishing LLC.; 2023.

[31] Lienhart A, Auroy Y, Péquignot F, Benhamou D, Warszawski J, Bovet M, Jougla E (2006). Survey of anesthesia-related mortality in France. Anesthesiology.

[32] Lee A, Festic E, Park PK, Raghavendran K, Dabbagh O, Adesanya A, Gajic O, Bartz RR (2014). Characteristics and outcomes of patients hospitalized following pulmonary aspiration. Chest.

[33] Schmitz A, Schmidt AR, Buehler PK, Schraner T, Fruhauf M, Weiss M, Klaghofer R, Kellenberger CJ (2016). Gastric ultrasound as a preoperative bedside test for residual gastric contents volume in children. Paediatr Anaesth.

[34] Perlas A, Mitsakakis N, Liu L, Cino M, Haldipur N, Davis L, Cubillos J, Chan V (2013). Validation of a mathematical model for ultrasound assessment of gastric volume by gastroscopic examination. Anesth Analg.

[35] Cho EA, Huh J, Lee SH, Ryu KH, Shim JG, Cha YB, Kim MS, Song T (2021). Gastric Ultrasound assessing gastric emptying of Preoperative Carbohydrate drinks: a Randomized Controlled Noninferiority Study. Anesth Analg.

[36] Shin HJ, Koo BW, Lim D, Na HS (2022). Ultrasound assessment of gastric volume in older adults after drinking carbohydrate-containing fluids: a prospective, nonrandomized, and noninferiority comparative study. Can J Anaesth.

[37] Tourkmani AM, Alharbi TJ, Rsheed AMB, AlRasheed AN, AlBattal SM, Abdelhay O, Hassali MA, Alrasheedy AA, Al Harbi NG, Alqahtani A (2018). Hypoglycemia in type 2 diabetes Mellitus patients: a review article. Diabetes Metab Syndr.

[38] Borzi V, Frasson S, Gussoni G, Di Lillo M, Gerloni R, Augello G, Gulli G, Ceriello A, Solerte B, Bonizzoni E (2016). Risk factors for hypoglycemia in patients with type 2 diabetes, hospitalized in internal medicine wards: findings from the FADOI-DIAMOND study. Diabetes Res Clin Pract.

[39] Lee DY, Han K, Park S, Yu JH, Seo JA, Kim NH, Yoo HJ, Kim SG, Choi KM, Baik SH (2020). Glucose variability and the risks of stroke, myocardial infarction, and all-cause mortality in individuals with diabetes: retrospective cohort study. Cardiovasc Diabetol.

[40] Cai Y, Zhang H, Li Q, Zhang P (2022). Correlation between blood glucose variability and early therapeutic effects after intravenous thrombolysis with alteplase and levels of serum inflammatory factors in patients with Acute ischemic stroke. Front Neurol.

[41] Oliveira CB, Garcia AKA, Nascimento LAD, Conchon MF, Furuya RK, Rodrigues R, Fonseca LF (2022). Effects of carbohydrate use on preoperative thirst: a randomized clinical trial. Rev Bras Enferm.

[42] Aroni P, Fonseca LF, Ciol MA, Margatho AS, Galvao CM (2020). The use of mentholated popsicle to reduce thirst during preoperative fasting: a randomised controlled trial. J Clin Nurs.

[43] Zachwieja JJ, Costill DL, Beard GC, Robergs RA, Pascoe DD, Anderson DE (1992). The effects of a carbonated carbohydrate drink on gastric emptying, gastrointestinal distress, and exercise performance. Int J Sport Nutr.

